# The impact of cervical conization size with subsequent cervical length changes on preterm birth rates in asymptomatic singleton pregnancies

**DOI:** 10.1038/s41598-021-99185-0

**Published:** 2021-10-05

**Authors:** Sergei V. Firichenko, Michael Stark, Ospan A. Mynbaev

**Affiliations:** 1 Moscow Institute of Physics and Technology (National Research University), Dolgoprudny, Moscow Region Russia; 2 Moscow State University of Medicine and Dentistry (Named After A.I.Evdokimov), Moscow, Russia; 3 The International Bureau of Human Body Design, Biomodeling & Biosensoring, Moscow, Russia; 4 The New European Surgical Academy (NESA), Berlin, Germany

**Keywords:** Preterm birth, Urogenital reproductive disorders, Diagnosis, Rehabilitation

## Abstract

The study aimed to explore the impact of cervical conization size (CCS) with subsequent cervical length (USCL) changes on preterm birth (PTB) rates in asymptomatic singleton pregnancies as compared to pregnancy outcomes in healthy women with an intact cervix (ICG), and to estimate PTB prevention efficiency in patients with a short cervix. Pregnancy outcomes in populations of similar age, ethnicity, residency, education and harmful habits having undergone cervical conization (CCG) were retrospectively analyzed and compared to ICG and cervical conization sub-populations adjusted by USCL during pregnancy (adequate cervical length vs. a short cervix) and a progesterone-only group (POG) vs. a progesterone-pessary group (PPG). Cervical conization was not associated with an increased PTB risk (CCG vs. ICG) when parameters of CCS and USCL were not adjusted (p = NS). A significantly higher proportion of parous women was observed in the CCG population than in the ICG (p = 0.0019). CCS turned out to be a key PTB risk during pregnancy, the larger CCS being associated with a short cervix (p = 0.0001) and higher PTB risks (p = 0.0001) with a notably increased PTB rate (p = 0.0001) in nulliparous women (p = 0.0022), whereas smaller CCS with adequate cervical length and a lower PTB rate was predominantly observed in women with prior parity. An initial equal USCL size was to be considerably elongated in women with adequate cervical length (p < 0.0001), and shortened in those with a short cervix (p < 0.0001). USCL assessment during pregnancy proved to be the PTB risk-predicting tool, with CCS supplementation apt to increase its diagnostic value. No substantial impact on pregnancy outcomes could be linked to any particular PTB prevention mode (POG or PPV). However, during pregnancy, the USCL changes relating to CCS proved to be more critical in pregnancy outcomes.

## Introduction

Preterm birth (PTB) is defined as any birth before 37 weeks of gestation or less than 259 days since the first day of a woman’s last menstrual period^1^. Due to a high neonatal mortality (about 1 million deaths)^1,2^ the estimated 15 million annual PTBs are a major worldwide healthcare concern. Furthermore, survivors may experience severe lifelong morbidity with a harmful impact on the individual, family, community and societal level^3^. Moreover, the need for and high costs of medical services and highly trained professionals are a financial burden on the healthcare and social systems in developed countries^1–3^. An expected increasing trend in global PTB prevalence is projected, based on overcoming of non-registration of preterm-born babies and the lacking subsequent statistical analysis in many low-income and developing countries^1–3^.

A traumatized and shortened cervix resulting from surgical procedures such as cold knife biopsy, conization, and excisions presented both a mechanical causative factor of PTB^4,5^ and acted as its predictor^6–8^. Therefore, many professional societies recommend transvaginal ultrasound cervical length (USCL) measurements to predict PTB risk^5,9^.

Historically, electrosurgical treatment of cervical pathologies has been suggested as a PTB risk factor^10–16^ whereas other studies have shown no impact on pregnancy outcomes^17–22^ thus leading to conflicting expert opinions^4,5,23^, being largely the result of the complexity of PTB causative mechanisms^24,25^.

In the special clinical guideline from the Society for Maternal–Fetal Medicine publications committee^26^, vaginal progesterone was recommended as a noninvasive PTB prevention for asymptomatic women with a short cervix whereas, since Cross^27^ published successful results of the use of a ‘Bakelite’ cervical ring to prevent habitual abortions due to cervical incompetence, pessaries have been used for over 60 years, modified and improved over time by Arabin^28^. Nowadays, both progesterone and pessaries are being used in the prevention of PTB in patients with a short cervix, although in literature the efficiency of these treatments has been extensively debated, yielding disputable results for vaginal progesterone^29–34^, cervical pessary^35–41^, and their inter-comparisons or combination^42–45^.

This study was designed to explore the impact of cervical conization size (CCS) with subsequent USCL changes on the PTB rates in asymptomatic singleton pregnancies compared to the pregnancy outcomes in women with an intact cervix and to estimate the efficiency of vaginal progesterone-only or in combination with a pessary to prevent PTB in patients with a short cervix.

## Results

**Figure 1:**
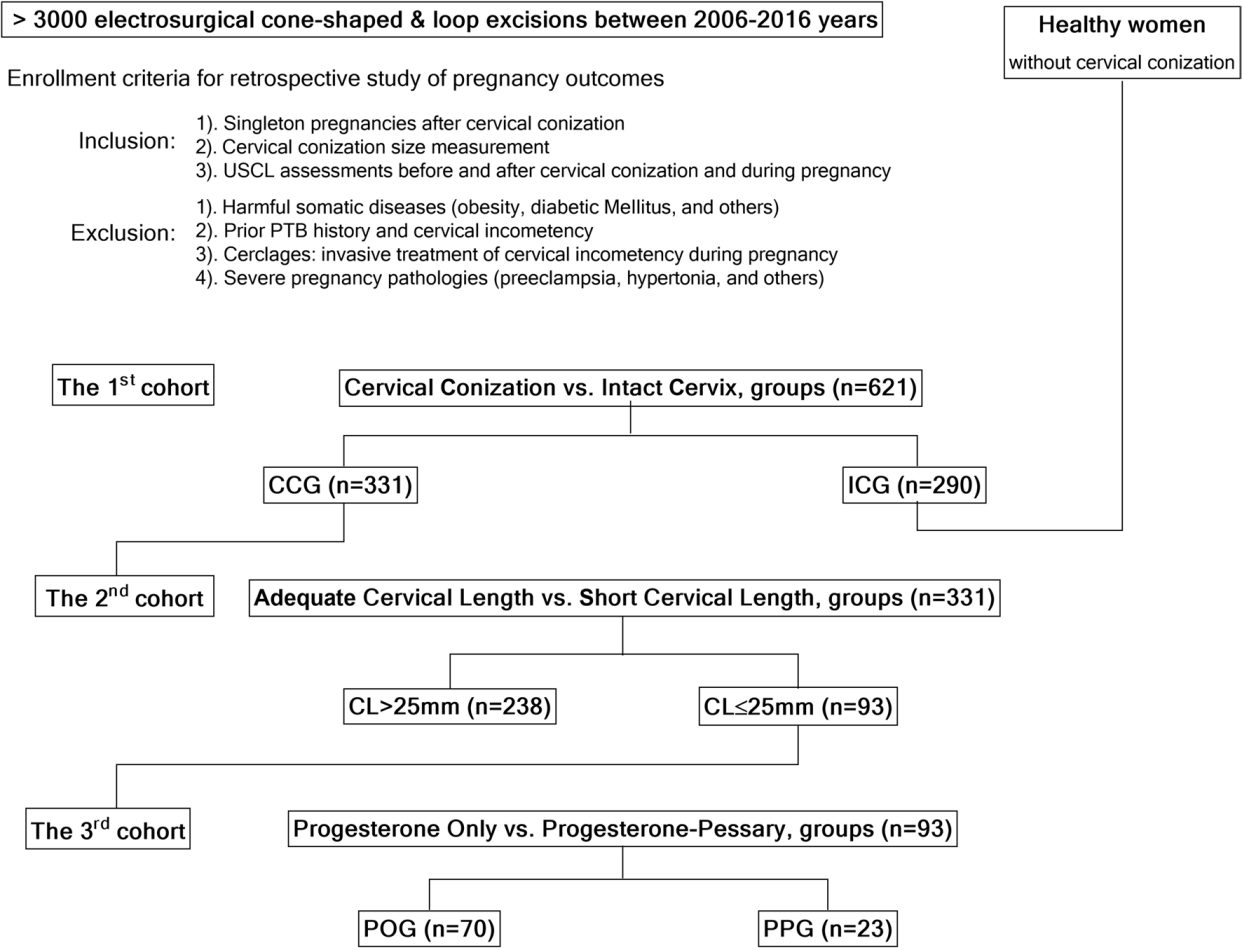
A flowchart of cervical conization impact on pregnancy outcome. *PTB* preterm birth, *USCL* ultrasound cervical length, *CCG* cervical conization group, *ICG* intact cervix group, *CL > 25 mm* adequate cervical length group, *CL ≤ 25 mm* short cervix group, *POG* Progesterone-only group, *PPG* Progesterone-Pessary group.

Several hypotheses were studied: the impact of cervical conization size (CCS), followed by ultrasound cervical-length changes (USCL) in asymptomatic, singleton pregnancies and the efficiency of different PTB prevention modalities in pregnancy outcomes in women with a short cervix. Consequently, women showing PTB risk factors were excluded whereas pregnant women with only the cervical factor after conization (CCG) were included in this retrospective study (Fig. 1). The pregnancy outcomes in healthy women with an intact cervix (ICG) served as a control group. Comparisons were performed in three steps with two groups to each cohort: in the 1st cohort (ICG, n = 290 vs CCG, n = 331); in the 2nd cohort—women with adequate cervical length (n = 238) vs a short cervix (n = 93), (CL > 25 mm vs. CL ≤ 25 mm); in the 3rd cohort—progesterone-only (n = 70) vs progesterone-pessary (n = 23) groups, (POG vs. PPG).

### Demographic factors

The cervical conizations followed by pregnancies occurred mostly in asymptomatic women in their late twenties. Most of these women were Moscow inhabitants from the Slavic ethnic population showing various levels of education (higher, professional, and secondary) and of which less than 10% were smokers (Table 1). Comparisons of all these factors showed no significant differences between the groups (ICG vs CCG, p = NS) in the 1st cohort. Subsequently, comparisons of variables in the groups (CL > 25 mm vs CL ≤ 25 mm, n = NS) and (POG vs PPG, n = NS) in the 2nd and 3rd cohorts demonstrated a similar demographic composition of the population in our study (Supplementary Tables 1 and 2).

**Table 1:** Demographic, obstetric, and pregnancy outcome parameters with values of mean and standard deviation (SD), 95% lower/upper confidential intervals (CI) of the mean in the 1st cohort: Intact Cervix (ICG) vs Cervical Conization (CCG), groups. p-values were achieved using the Mann–Whitney two-tailed t-test with Alpha = 0.05.

Parameters	Groups				p values
	ICG, n = 290		CCG, n = 331		
	Mean, SD	95% CI	Mean, SD	95% CI	
Age, years	28.33 ± 5.03	27.75/28.92	28.03 ± 5.74	27.41/28.65	NS
Moscow residency, %	0.91 ± 0.29	0.88/0.94	0.92 ± 0.26	0.89/0.95	NS
Slavic ethnicity, %	0.84 ± 0.36	0.80/0.89	0.86 ± 0.34	0.83/0.90	NS
Higher education, %	0.31 ± 0.46	0.26/0.36	0.32 ± 0.47	0.27/0.37	NS
Professional education, %	0.38 ± 0.49	0.32/0.43	0.38 ± 0.48	0.32/0.43	NS
Secondary education, %	0.31 ± 0.46	0.28/0.29	0.30 ± ± 0.46	0.27/0.29	NS
Smoking population, %	0.10 ± 0.30	0.06/0.13	0.07 ± 0.26	0.04/0.10	NS
Conization-pregnancy interval, months	–	–	12.85 ± 5.32	12.27/13.42	–
Cesarean section, %	0.20 ± 0.40	0.16/0.25	0.19 ± 0.39	0.15/0.24	NS
Vaginal birth, %	0.80 ± 0.40	0.75/0.84	0.81 ± 0.39	0.76/0.85	NS
Spontaneous amniotic membrane ruptures, %	0.11 ± 0.31	0.07/0.14	0.13 ± 0.34	0.10/0.17	NS

### Cervical conization size (removed cervical tissue volume)

**Figure 2:**
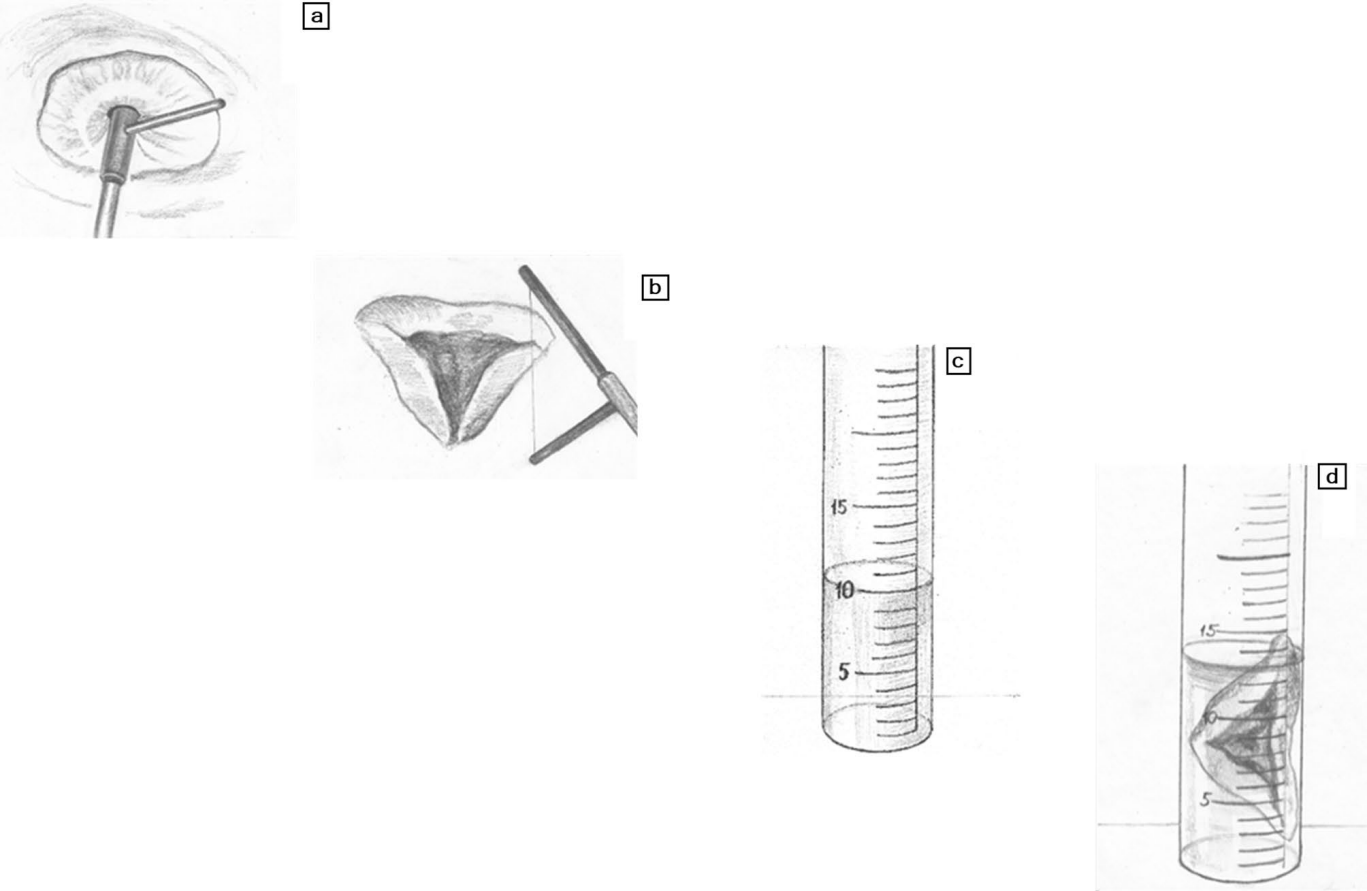
Assessment of the volume of the removed sample, defined as the cervical conization size (unit: cm^3^): (**a**) conization of the pathologic part of the cervix; (**b**) removed sample; (**c**) a syringe with 10 ml water; (**d**) estimation of the volume of the removed sample in ml^3^. The authors gratefully acknowledge Iulia V. Chumarina (Firichenko).

**Figure 3:**
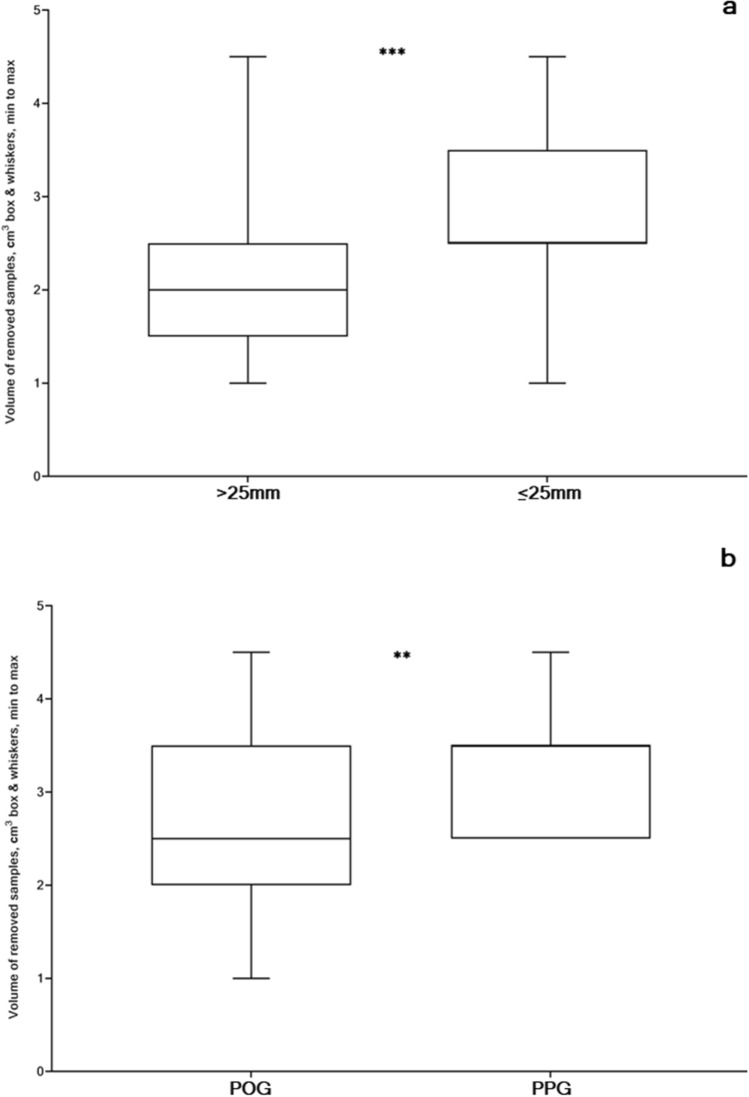
Cervical conization size (unit: cm^3^, box & whiskers, min to max) in asymptomatic women with singleton pregnancies: (**a**) Adequate cervical length (CL > 25 mm) vs Short cervix (CL ≤ 25 mm), groups; (**b**) Progesterone-only (POG) vs Progesterone-Pessary (PPG), groups. p-values were achieved using Mann–Whitney two-tailed t-test with Alpha = 0.05.

CCS (cm^3^) was achieved during conization (Fig. 2a–d), and mean values with standard deviations and 95% confidential intervals of the mean (CI) were analyzed (Fig. 3a,b): CL > 25 mm 2.16 cm^3^ with 95% CI (2.06–2.26); CL ≤ 25 mm 2.88 cm^3^ with 95% CI (2.7–3.1); POG 2.73 cm^3^ with 95% CI (2.52–2.93); PPG 3.33 cm^3^ with 95% CI (3.01–3.64) with a mean size of CCS in CCG 2.36 cm^3^ with 95% CI (2.27–2.46). A significantly small CCS was observed in the CL > 25 mm group as compared to the CL ≤ 25 mm group (p < 0.0001). Analogously, significantly small CCS was found in the POG rather than in the PPG (p = 0.0030). These results demonstrate that CCS is an important factor in future pregnancy outcomes in patients with changing cervical length, the bigger CCS possibly resulting in a cervix shortening.

### Ultrasound cervical length changes during pregnancy in women with and without cervical conization and depending on PTB prevention modalities

**Figure 4:**
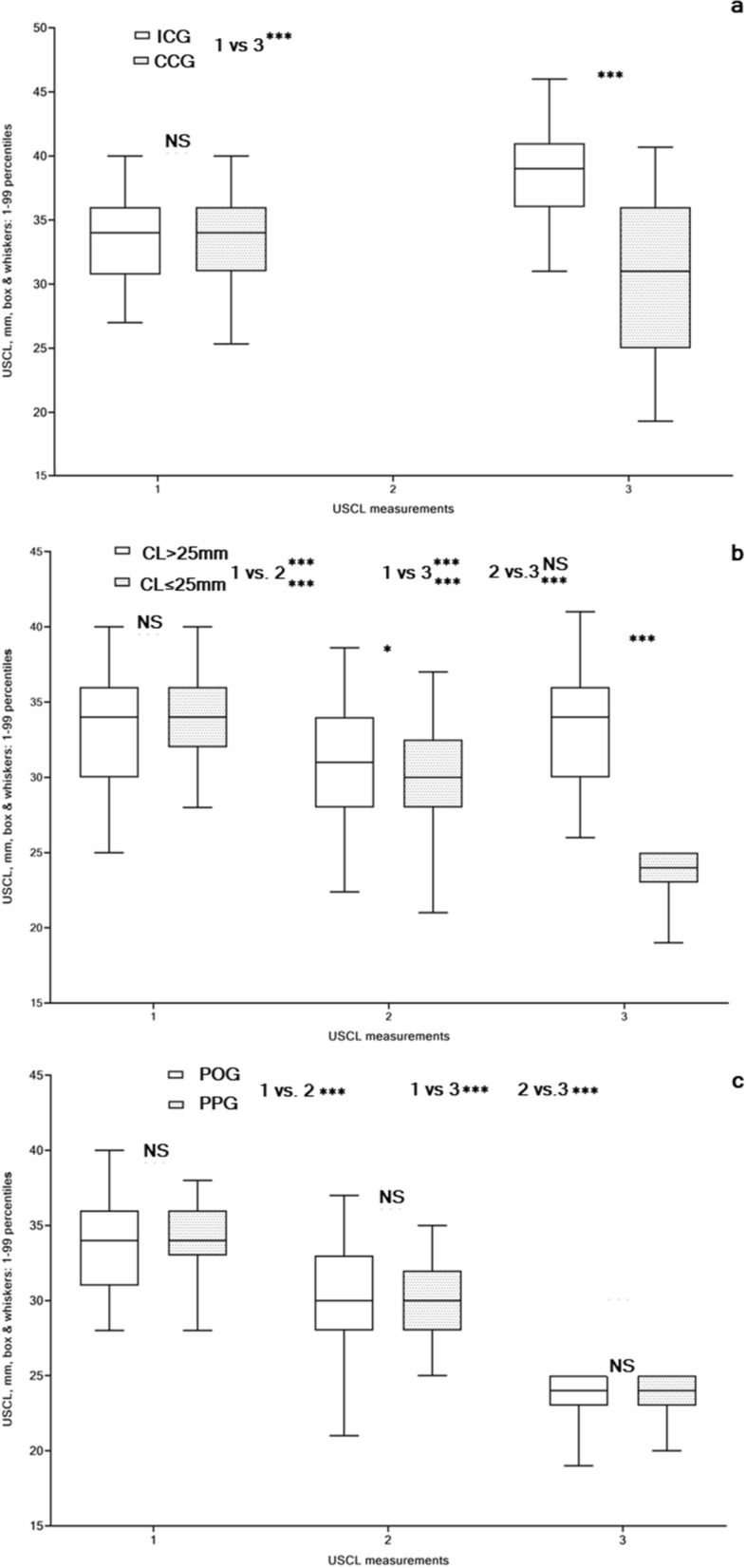
Ultrasound cervical length (USCL) assessed in non-pregnant women before (1) and after (2) cervical conization, and during pregnancy (3), unit: mm, box & whiskers, 1–99 percentiles) in asymptomatic women with singleton pregnancies: (**a**) Intact Cervix (ICG) vs. Cervical Conization (CCG), groups; (**b**) Adequate cervical length (CL > 25 mm) vs Short cervix (CL ≤ 25 mm), groups; (**c**) Progesterone-only (POG) vs Progesterone-Pessary (PPG), groups.

In the 1st cohort, ultrasound cervical length (USCL) measurements—performed twice-before conization (1) and in a follow-up during pregnancy (3) and their respective results were analyzed with the one-way ANOVA with Tukey’s multiple comparisons test (Fig. 4a). Similar USCL parameters before conization (1 vs 1, p = NS) significantly changed during pregnancy (3 vs 3, p < 0.0001). All comparisons (1 vs 3) in both groups demonstrated significant differences between USCL measurements before and during pregnancy in both populations with the intact and the treated cervix (p < 0.0001). During pregnancy, USCL was significantly longer in women with an intact cervix, whereas USCL was shortened in women who experienced cervical conization as compared to the initial USCL measurements before conization (p < 0.0001).

In the 2nd and 3rd cohorts, USCL measurements were performed thrice (Fig. 4b,c), before (1) and after (2) conization, and during pregnancy (3), and results were analyzed with the two-way ANOVA statistics and Bonferroni’s multiple comparisons test. The impact of measurement time on USCL changes in the 2nd cohort comparisons was highly significant (p < 0.0001). Both the impact of USCL and the impact of measurement time on the overall results (the curves) were highly significant (p < 0.0001). Both subject impact and interactions were highly significant (p < 0.0001). USCL values in both women with an adequate and a short cervix before conization were similar (1 vs 1, p = 0.1630) but significantly changed after conization (2 vs 2, p = 0.0174) and during pregnancy (3 vs 3, p < 0.0001). USCL in women with CL > 25 mm significantly shortened after conization (1 vs 2) (p < 0.0001) and USCL significantly lengthened during pregnancy (2 vs 3) (p < 0.0001) reaching the initial length (1 vs 3) (p = NS). Dramatically shortened USCL in women with CL ≤ 25 mm was observed in both follow-up measurements after conization (1 vs 2) and during pregnancy (1 vs 3), as well as in (2 vs 3) (p < 0.0001).

There was no significant USCL impact on the overall results (the curves) and interactions in the 3rd cohort comparisons. The impact of measurement time on USCL changes was highly significant (p < 0.0001) as was the subject impact. All post hoc comparisons in women with short cervix having received POG and PPG (1 vs 1; 2 vs 2; 3 vs 3) were not significantly different (p = NS), although USCL follow-up parameters before and after conization (1 vs 2) and during pregnancy (1 vs 3 and 2 vs 3) were significantly shortened in both groups (p < 0.0001).

### Obstetrical factors

**Figure 5:**
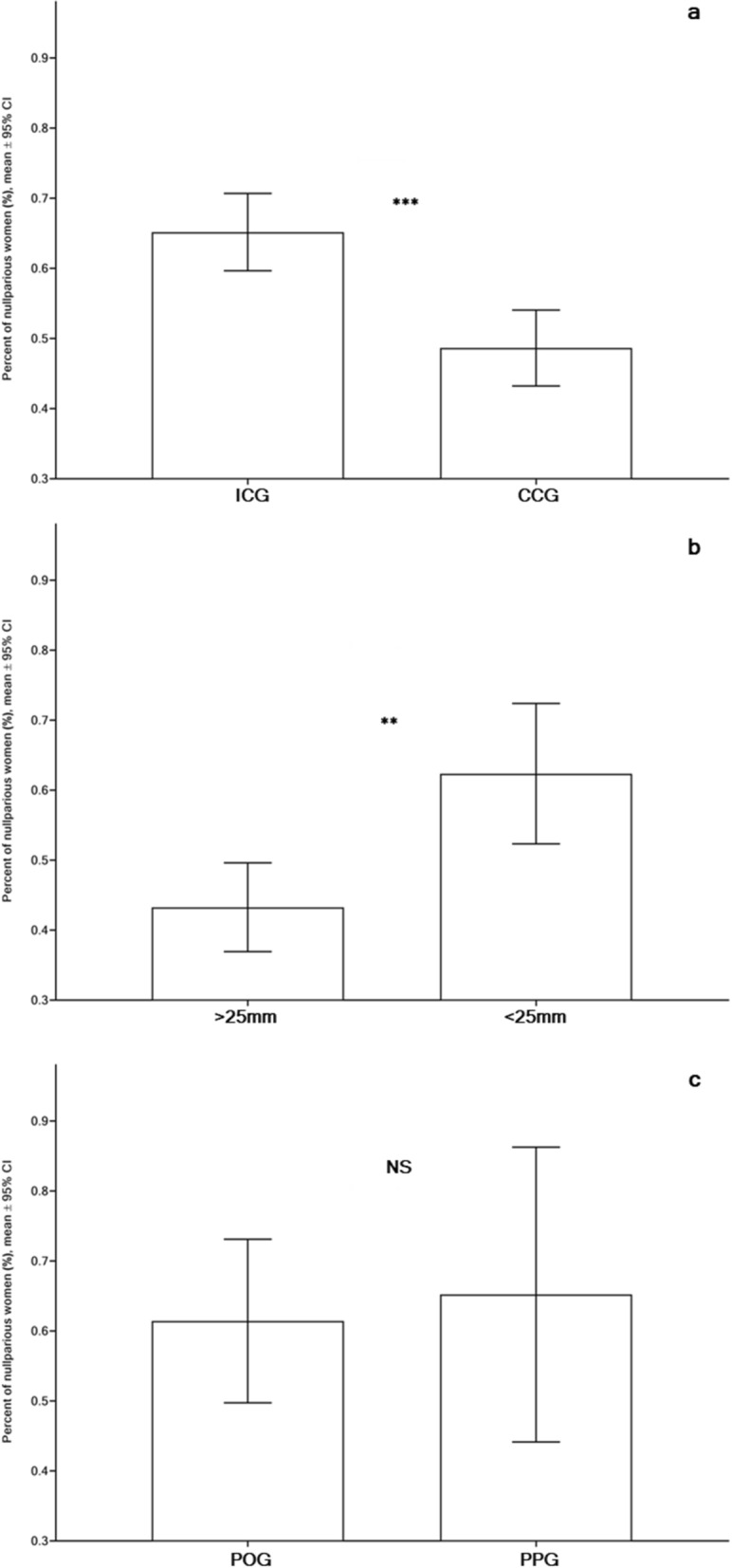
The rate of nulliparous women (unit: %, mean with 95% CI) in asymptomatic women with singleton pregnancies: (**a**) Intact Cervix (ICG) vs. Cervical Conization (CCG), groups; (**b**) Adequate cervical length (CL > 25 mm) vs Short cervix (CL ≤ 25 mm), groups; (**c**) Progesterone-only (POG) vs Progesterone-Pessary (PPG), groups. p-values were achieved using Mann–Whitney two-tailed t-test with Alpha = 0.05.

A significantly higher proportion of the nulliparous population (p < 0.0001) was observed in the ICG (65.17% with 95% CI 59.66–70.69) than that in the CCG (48.64% with 95% CI 43.23–54.05) supporting the physicians’ decisions to avoid performing cervical conizations in nulliparous women (Fig. 5a). On the contrary, a significantly higher proportion of parous women (PW) was observed in the CCG population than in the ICG (p = 0.0019).

The proportion of the nulliparous population in the CL > 25 mm group (43.28% with 95% CI 36.94–49.62) was significantly lower (Fig. 5b) in comparison to similar proportions of the CL ≤ 25 mm group (62.37% with 95% CI 52.33–72.4) (p = 0.0022), showing that nulliparous women having experienced cervical conization are susceptible to shortening of the cervix during pregnancy. Equal proportions of nulliparous women were observed in the POG and PPG (Fig. 5c).

In total, the intervals between cervical conization and conception lasted 12.85 ± 5.32 months with 95% CI (12.27–13.42) in the CCG (Table 1). There were significantly shorter intervals (p = 0.0179) in women with CL > 25 mm 12.28 months with 95% CI (11.69–12.87) than in those with CL ≤ 25 mm 14.30 months with 95% CI (12.94–15.66) respectively (see Supplementary Table 2).

There was a similar Cesarean section rate in healthy women and in those who had undergone cervical conization, respectively in 20% with 95% CI (16–25%) and 19% with 95% CI (15–24%) of cases (see Table 1). The highest 24% with 95% CI (14–35%) and the lowest rates 17% with 95% CI (10–34%) were observed respectively in the POG and PPG without significant differences (p = NS). Subsequently, none of the three cohorts showed significant differences in vaginal birth rates (p = NS).

Spontaneous membrane rupture was observed in 10.69% with 95% CI (9.33–9.87) cases in the ICG and 7.84% with 95% CI (7.59–8.09) cases in the CCG with similar rates in the compared groups of the other two cohorts (see Table 1 and Supplementary Tables 1 and 2).

Possibly as a result of having excluding the PTB risk factors, all deliveries started spontaneously. Births lasted 9.6 h with 95% CI (9.33–9.87) in the ICG (n = 250) showing a significantly longer time than in the CCG (n = 141) 7.84 h with 95% CI (7.59–8.09), (p < 0.0001).

In women with cervical conization, birth lasted equally 7.83 h with 95% CI (7.5–8.16) in CL > 25 mm (n = 90) and 7.84 h with 95% CI (7.46–8.23) in CL ≤ 25 mm (n = 51). Analogously, 7.76 h with 95% CI (7.32–8.2) in the POG (n = 41) and 8.2 h with 95% CI (7.32–9.08) in the PPG (n = 10) with no significant differences in all comparisons (p = NS).

**Figure 6:**
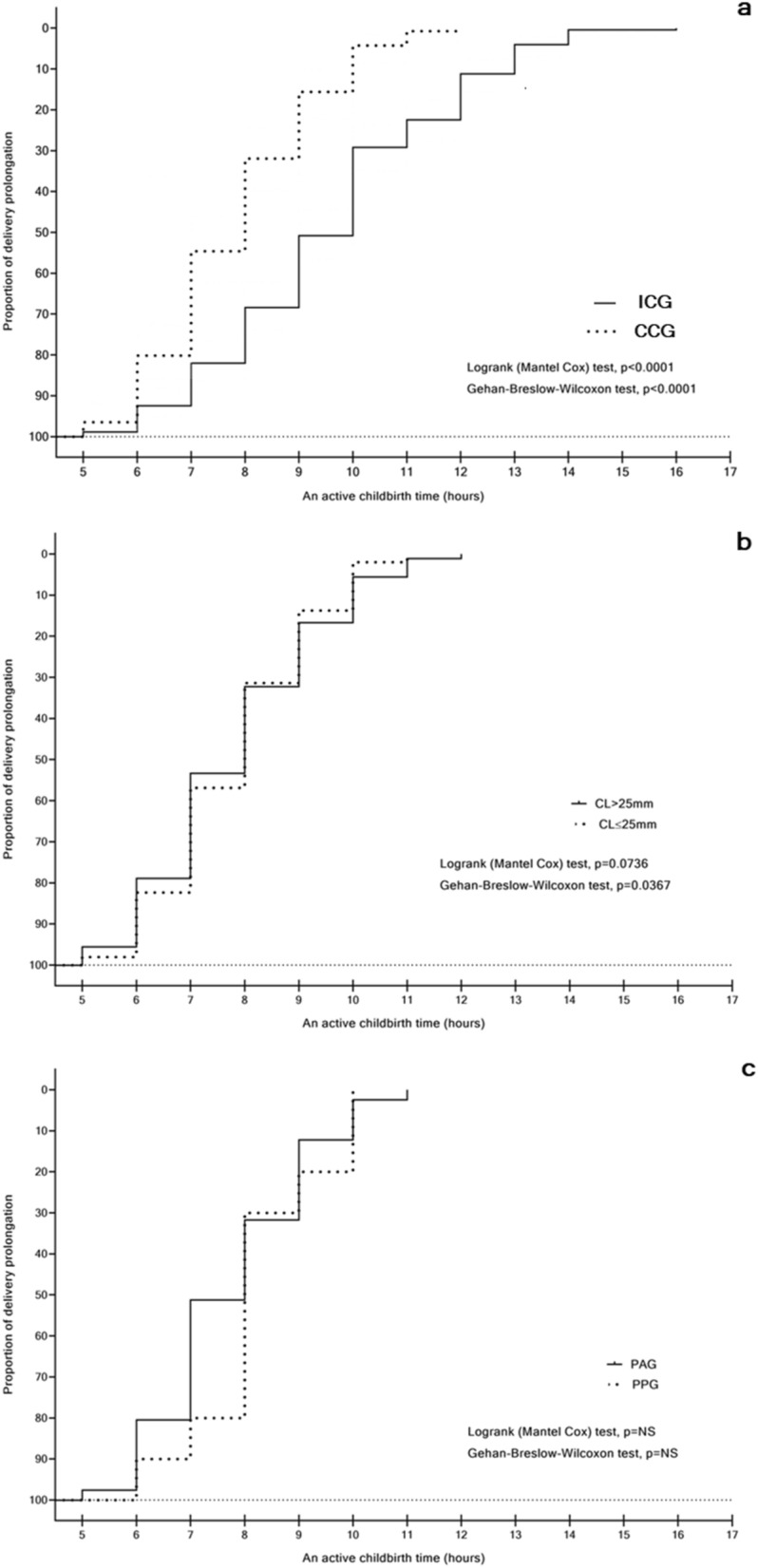
The delivery prolongation curve, (unit: survival curve in hours of the proportion of delivery prolongation) in asymptomatic women with singleton pregnancies: (**a**) Intact Cervix (ICG) vs. Cervical Conization (CCG), groups; (**b**) Adequate cervical length (CL > 25 mm) vs Short cervix (CL ≤ 25 mm), groups; (**c**) Progesterone-only (POG) vs Progesterone-Pessary (PPG), groups.

However, in different time intervals, the survival curve analysis demonstrated substantial differences of proportions of delivered women between the groups compared in the 1st cohort for both tests (p < 0.0001) and in the 2nd cohort for the Gehan–Breslow–Wilcoxon test (p = 0.0367) and logrank (Mantel Cox) test (p = 0.0736) (Fig. 6a–c). There were no significant differences in these proportions between the POG and PPG (p = NS).

### Endpoints

In the ICG, pregnancies lasted 39.1 with 95% CI (38.94–39.25) and in the CCG, 39.07 with 95% CI (39.87–39.26). Analogous pregnancy durations were found in other comparisons: in CL > 25 mm 39.34 with 95% CI (39.18–39.5) and CL ≤ 25 mm, 38.37 with 95% CI (37.82–38.91) weeks, as well as in the POG 38.6 with 95% CI (38.02–39.18) and the PPG 37.74 with 95% CI (36.36–39.12). There were no significant differences between the compared groups in all three cohorts (p = NS) where a non-parametric two-tailed t-test was applied.

**Figure 7:**
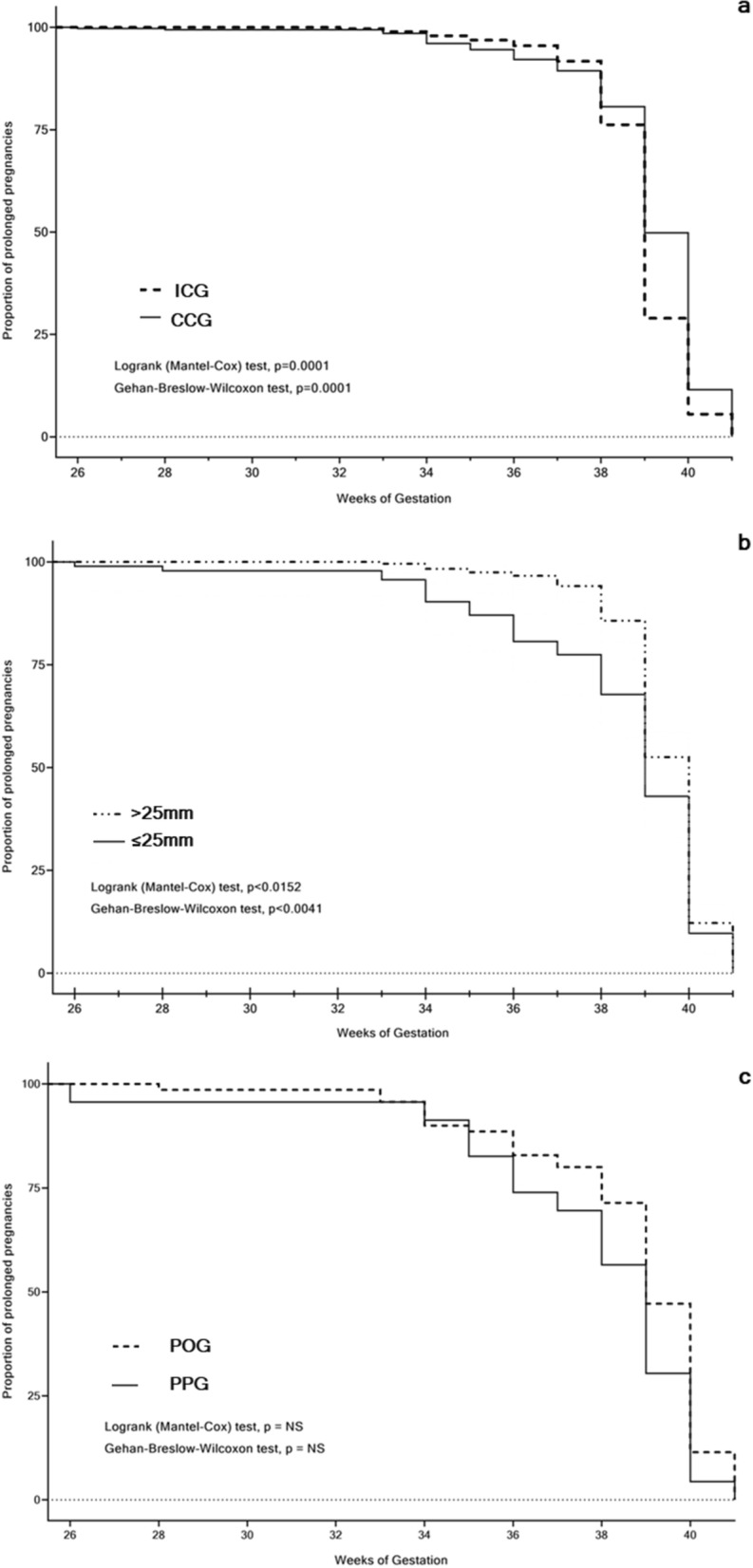
The pregnancy duration curve (unit: the proportion of prolonged pregnancies during 26–41 weeks) in asymptomatic women with singleton pregnancies: (**a**) Intact Cervix (ICG) vs. Cervical Conization (CCG), groups; (**b**) Adequate cervical length (CL > 25 mm) vs Short cervix (CL ≤ 25 mm), groups; (**c**) Progesterone-only (POG) vs Progesterone-Pessary (PPG), groups.

Further, pregnancy prolongation proportion was analyzed using the survival curve (logrank tests Mantel Cox and Gehan–Breslow–Wilcoxon) analysis (Fig. 7a–c).

There were significantly higher proportions of pregnancy prolongation between 32 and 37 weeks of gestation in the ICG as compared to the CCG (p < 0.0001) for both tests. The lower proportions of pregnancy prolongation were observed in women with CL ≤ 25 mm as compared to those with CL > 25 mm (p < 0.0152 and p < 0.0041, respectively survival tests). The proportions of pregnancy prolongation did not significantly change in women who received PTB prevention treatments (POG vs PPG, p = NS).

**Figure 8:**
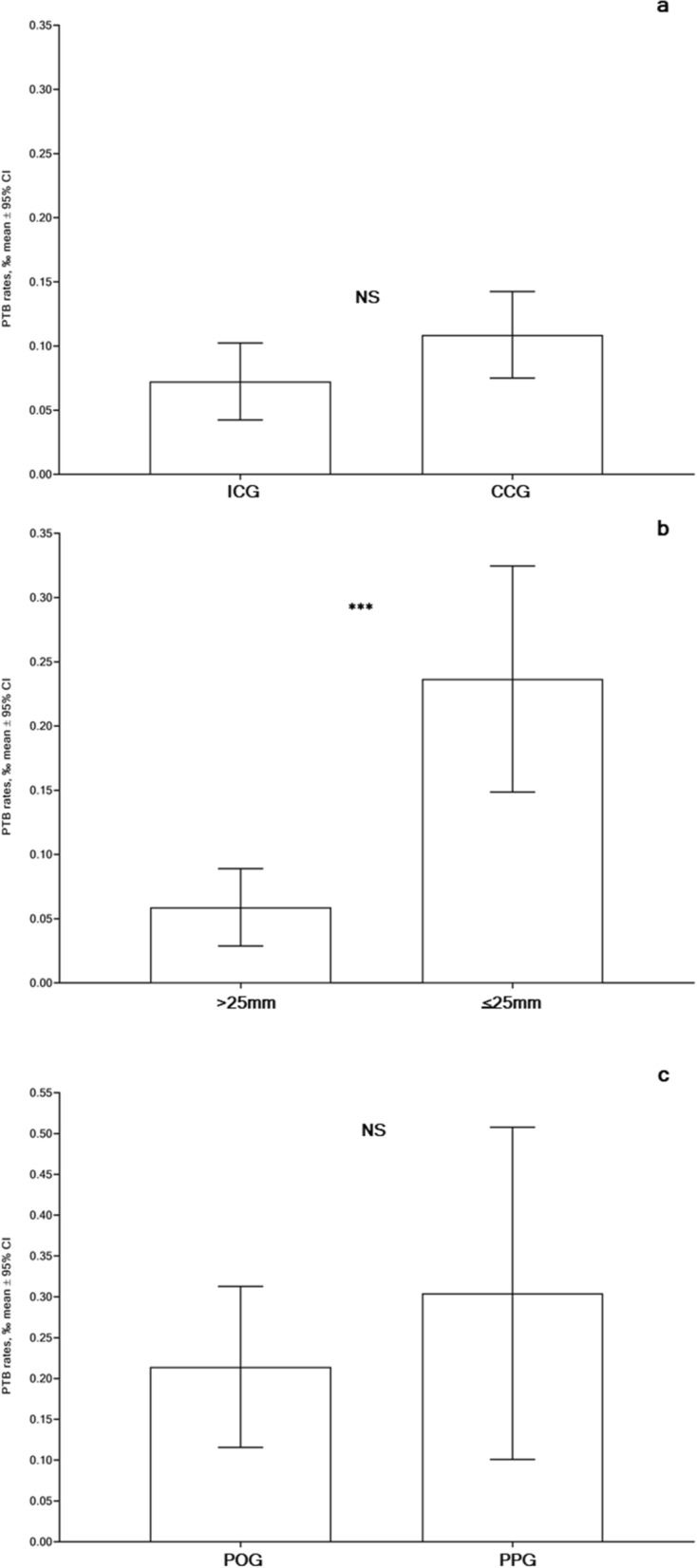
Preterm birth (PTB) rates (unit: %) in asymptomatic women with singleton pregnancies: (**a**) Intact Cervix (ICG) vs. Cervical Conization (CCG), groups; (**b**) Adequate cervical length (CL > 25 mm) vs Short cervix (CL ≤ 25 mm), groups; (**c**) Progesterone-only (POG) vs Progesterone-Pessary (PPG), groups. p-values were achieved using Mann–Whitney two-tailed t-test with Alpha = 0.05.

Finally, PTB rates in the ICG were 7.24% with 95%CI (4.24–10.24%) and in the CCG—10.88% with 95% CI (7.5–14.24%), with no significant differences between these groups (p = NS) (Fig. 8a–c). The PTB rate in group CL > 25 mm was 5.88% with 95% CI (2.87–8.89%), whereas in CL ≤ 25 mm estimated 23.66% with 95% CI (14.86–32.46%) with highly significant differences (p < 0.0001). PTB rate in the POG was 21.43% with 95% CI (11.57–31.28%) and in the PPG increased up to 30.43% with 95% CI (10.09–50.78%) but there were no significant differences in this comparison (p = NS).

### Relative risks and odds ratios

Significantly increased risk ratios, such as PTB relative risk 0.51 with 95% CI (0.32–0.73) and odds ratio 0.20 with 95% CI (0.09–0.41) were found between women with a short cervix and those with an adequate cervical length (p = 0.0001).

## Discussion

Firstly, the study retrospectively analyzed the pregnancy outcomes of asymptomatic women of similar age, ethnicity, residency, educational background and harmful habits (smoking) of populations in an intact cervix group (ICG) vs. those in a cervical conization group (CCG). In a further two steps, the pregnancy outcomes of women having undergone cervical conization were analyzed depending on USCL adjustment (CL > 25 mm vs. CL ≤ 25 mm) and PTB prevention modes (POG vs. PPG). Medical records of women with somatic diseases, obesity, and severe pregnancy pathologies were excluded to limit PTB co-morbidity factors and to focus on the impact of conization on pregnancy outcomes.

Cervical conizations followed by pregnancies were performed mostly in asymptomatic women in their late twenties, this being the age group showing a peak in severe dysplasia incidence^46^.

There was a significantly lower nulliparous proportion in the women who had undergone cervical conization in comparison to women without cervical conization (p < 0.0001), supporting the physicians’ decisions to avoid cervical conizations in nulliparous women. These findings were in accordance with the lower number of nulliparous vs. parous populations (19.2% vs. 80.8%) in women having had histopathological examinations of cervical samples after conizations^47^. This may also explain our observation concerning the significantly reduced duration of the active labour phase in women who had experienced cervical conization than in women with an intact cervix (p < 0.0001) since a shorter delivery prolongation was observed in parous women as compared to in the nulliparous population^48^. However, a significantly higher proportion of deliveries occurred at the beginning of the process in women with a short cervix (CL ≤ 25 mm) in comparison to women with adequate cervical length (CL > 25 mm), achieved using the survival trend Gehan–Breslow–Wilcoxon test (p = 0.037). Our findings, showing reduced delivery time in women with cervical conization, contradicted the data showing prolonged delivery time in women with cervical conizations vs. those without^11^. It is well-known, that both shortened or prolonged labour might have potentially harmful consequences on both the newborn’s and the mother’s health^49,50^. Consequently, deliveries in women with cervical conization need further study.

The Cesarean section and vaginal birth rates, as well as the spontaneous amniotic membrane rupture rates, were similar in the compared populations. Our findings showed a similarity in spontaneously ruptured amniotic membrane rate in the compared groups in all the three cohorts and contradict literature data demonstrating increased amniotic membrane ruptures in women with cervical conizations vs. those without^51,52^ and a link between amniotic membrane ruptures and impending preterm birth^53^. The explanation of these contradictory findings might lie in the exclusion of the PTB risk factors in our study and the possibly confounding impact of different types of used conization techniques in previous observations^54^.

Our comparisons of the PTB rates and pregnancy duration in women, both with and without cervical conization in the 1st cohort, when the USCL was not adjusted, were similar to the literature data, showing no differences between these groups^17,19,22^ and contradicted comparisons in similar (treated vs. nontreated) populations^54^. However, the authors speculated that the different types of cervical excision methods used in their meta-analysis were related to the increased PTB rates^54^. They also mentioned that PBT rates were less affected by LEEP than by cold knife conization^54^. Our findings may therefore shed light on this relationship since only electrosurgical cone-shaped and loop probes were used.

PTB rate risks (relative risk and odds ratio) significantly increased in women with a short cervix (CL ≤ 25 mm) in comparison to women with adequate cervical length (CL > 25 mm) (p < 0.0001). Consequently, we can confirm that the study results demonstrating the controversial impact of cervical electrosurgical procedures on PTB rates^4,5^ in comparison to pregnancy outcomes in external or internal controls^54^ are dependent on CCS^13,53,55–57^ and USCL adjustment^5,6,58^.

The survival curve analysis demonstrated that, in this study, pregnancy duration in short-cervix women (CL ≤ 25 mm) was similar to the findings of Iams et al.^6^ who compared two analogous groups adjusted for USCL ≤ 25 vs > 25 mm. We found similar points of view in literature, where increased PTB rates in women with short cervix after cervical conization remained after adjusting for maternal age, parity, and smoking^59^, and prior PTB history^60^. These results showed that after cervical electrosurgical procedures, USCL is the cornerstone in pregnancy outcome. Consequently, USCL changes before and during pregnancy are to be thoroughly analyzed due to the high clinical importance of these findings.

In the 1st cohort, USCL values before pregnancy were similar (ICG vs. CCG), whereas during pregnancy this parameter significantly changed in both groups (p < 0.0001). During pregnancy, USCL was significantly longer in women with an intact cervix, whereas USCL was shorter in women who had experienced cervical conization as compared to those in the initial USCL before conization (p < 0.0001). In the 2nd cohort, USCL values in women both with an adequate and a short cervix before conization were similar and significantly changed after conization (p = 0.0188) and during pregnancy (p < 0.0001). USCL in women with an adequate cervix significantly shortened after conization (p < 0.0001) and then elongated during pregnancy (p < 0.0001) with reaching the initial length (USCL: before conization = during pregnancy). Dramatically shortened USCL in women with a short cervix was observed in both the follow-up measurements after conization and during pregnancy (p < 0.0001). Analogously, USCL follow-up parameters in women who received PTB prevention treatments were significantly shortened after conization and during pregnancy in comparison to the initial values before conization in both groups (p < 0.0001).

Our results regarding PTB rates and pregnancy duration in women with a short cervix receiving vaginal progesterone-only were similar with findings^32,33^ and contradicted^30,31,34^, analogously combined progesterone and pessary application outcomes were in agreement^42,45^ and contradicted^29,44,61^ as well as pessary alone in accord^38,40,41^ and contradicted^28,35,37^ with findings of publications using analogous PTB prevention modes. Our observation concerning the efficient similarity of both PTB prevention modes is in accord with literature^42,62^, although CCS in PPG was significantly bigger than that in POG (p = 0.0030). Furthermore, pronounced changes in parameters of USCL during pregnancy (see Fig. 4c), proportions of delivered women (see Fig. 6c) and proportions of prolonged pregnancies (see Fig. 7c), and PTB rates (see Fig. 8c) were observed among women in PPG than those in POG without significant differences between these groups (p = NS). It can be presumed that women in PPG were willing more attention and received additional pessary applications.

Significantly smaller CCS was observed in the CL > 25 mm than in the CL ≤ 25 mm (p < 0.0001). Analogously, significantly smaller CCS was found in the POG than in the PPG (p = 0.0030). These results demonstrate that CCS is an important factor in future pregnancy outcomes with changing cervical length; the bigger CCS possibly resulting in a cervix shortening. These findings are in accordance with analogous observations where the size (depth) of removed cervical tissue was taken into account to predict PTB rates^14,15,55–57^.

Generally, significantly higher PTB rates were observed in women with a short cervix in comparison to women with adequate cervical length (p = 0.0001). Analogously, substantially increased risk ratios, such as PTB relative risk and PTB odds ratio, were found in women with a short cervix vs in those with adequate cervical length (p = 0.0001).

It seems that a combination of several factors such as larger CCS and shortened USCL at 16–18 weeks of gestation depending on prior parity history (nulliparous or parous) plays a crucial role in PTB rate in asymptomatic women with singleton pregnancies. This CCS impact on PTB rates might be related to a high concentration of neuropeptides, neurotransmitters, oxytocin, and other physiologically active substances in the cervical tissue and lower uterine segment rather than to that in tissues in the upper part of the uterus^63,64^. By its modification, the cervix plays an important role during pregnancy and delivery as does the part of the uterine body forming the lower uterine segment^65,66^. Subsequently, we suggest that excessive cervical conization might lead to tissue insufficiency and impair cervix structure and function.

### Strengths

The study retrospectively analyzed singleton pregnancy outcomes in asymptomatic women in similar populations regarding age, national compositions, residency, background education levels and harmful habits (smoking). All pre-pregnancy cervical electrosurgical procedures, CCS measuring as well as most USCL assessments, were performed by trained professionals using the same equipment and instrumentation. Next, the statistical analysis of all results was performed by an independent researcher. The medical records of women with somatic diseases, obesity, and severe pregnancy pathologies were excluded so as to limit PTB co-morbidity factors and focus on the impact of conization size and cervical length changes on pregnancy outcomes. Three-step statistics in three cohorts enabled us to demonstrate significant USCL measurement values before and after cervical conization and during pregnancy as well as the estimated PTB prevention mode efficiency, i.e. vaginal progesterone-only and in combination with a pessary in women with a short cervix thus adjusting removed tissue size and the USCL changes before and during pregnancy. For the first time, observations of the USCL changes were monitored before and after conization in non-pregnant women and during pregnancy, supplemented by the simple method of a physical volume (size) assessment of the removed part of the cervix. Additionally, the findings for the lowest proportion of the nulliparous women with a short cervix as opposed to the highest number of parous women with adequate cervical length were described.

These results may contribute to understanding controversial PTB rates depending on the CCS and USCL changes in the general population of women after cervical conization compared to the control group of women without pre-pregnancy cervical conization. They may also allow a better understanding of the different PTB prevention modes. We suggest that during pregnancy the diagnostic and predicting value of the USCL assessment be implemented if CCS is to be taken into account, which can be the important point of view of this study for practitioners.

### Limitations

Due to the retrospective study design, our study has its limitations: the small number of patients in the group with combined application of progesterone and pessary, as well as the use of two pessary models might lead to a decreased statistical significance of our analysis. The comparison bodies for the PTB prevention modes were populations with an intact cervix without progesterone use and women with a short cervix who experienced cervical conization and who received vaginal progesterone during pregnancy. However, we could not provide the group with analogous short cervix without treatment, the placebo, or the pessary-use only group because of obvious ethical reasons and the necessity to follow the PTB management guidelines issued by the Healthcare Ministry of the Russian Federation. We believe in a necessity of further comparative studies of these PTB prevention modes. Women were delivered in different maternity houses and medical centers in Moscow, but with relatively similar quality of services, although this might be considered a limitation of our study. The retrospective nature of the study hampered our ability to collect data concerning newborns.

## Conclusions

This study may shed some light on the conflicting conclusions concerning the duplicity impact of cervical conization on pregnancy outcomes. The PTB risk was nullified in comparisons of populations with cervical conization and in those with an intact cervix when the USCL was not taken into account, resulting in somewhat provocative misleading results. The key point being that PTB risks depend on CCS. Furthermore, during pregnancy, the larger CCS was associated with a short cervix and higher PTB risks, notably increased in nulliparous women whereas the smaller CCS followed by adequate USCL and lower PTB risks were predominantly observed in women with prior parity. USCL assessment was the PTB risk predicting tool, possibly supplemented with CCS to increase its diagnostic value. There was no substantial impact on pregnancy outcome associated with the PTB prevention modes, whereas the USCL changes during pregnancy resulting from CCS were more critical for pregnancy outcomes.

## Materials and methods

### Study design and subjects

This retrospective cohort study was designed in the outpatient unit of the university obstetrics and gynecology hospital of the Moscow State University of Medicine & Dentistry (MSUMD) named after A.I.Evdokimov. The study protocol was approved by the institutional Ethics Committee of the MSUMD (№ 06-14/19.06.2014).

In the university outpatient clinic, more than 3000 electrosurgical cone-shaped (CONE) and loop (LEEP) excisions were performed over a 10-year period (2006–2016) in which we followed cervical pathologies, including human papillomavirus-related cervical intraepithelial neoplasia (CIN) grades 2–3 lesions and a combination of CIN grade 1–3 with ectropion or deformation of the cervix after previous births. There were no severe complications of these outpatient electrosurgical procedures concerning bleeding, infection, cervical stenosis and such upon required hospitalization or other urgent treatments. All diagnostic and treatment methods were carried out following good clinical practice guidelines, national diagnostic and treatment standards, and the regulations of the Russian Federation Health Care ministry with signed informed consent by the patients. The enrollment of the medical records of asymptomatic women with pregnancy outcomes was realized in accordance with the following criteria for inclusion: (1) Singleton pregnancies with a history of single cervical conization before the pregnancy; (2) Cervical conization size assessment according to our previously described method^67^; (3) The transvaginal ultrasound cervical length (USCL) assessments before and after cervical conization and during pregnancy. The USCL measurements during pregnancy were performed in asymptomatic women without miscarriage (PTB) symptoms (uterine contractions, pelvic pressure, a change in vaginal discharge^68^ and with these exclusion: (1) Harmful somatic diseases, obesity, diabetic Mellitus, and others; (2) Prior PTB history and cervical incompetency; (3) Cerclages—invasive treatment of cervical incompetency during pregnancy; (4) Severe pregnancy pathologies, (preeclampsia, hypertonia, and others).

Following these enrollment criteria, 331 medical records were found with pregnancy outcomes of women who had had cervical conizations (see Fig. 1). Additionally, medical records of singleton pregnancy outcomes of 290 healthy asymptomatic women without prior history of cervical conizations (intact cervix) and PTB, as well as with USCL assessments before and during pregnancy were collected. Finally, 621 pregnancy outcomes were analyzed in the 1st cohort and grouped: the intact cervix group (ICG, n = 290); cervical conization group (CCG, n = 331). Then women with cervical conizations were considered as the 2nd cohort, divided depending on USCL measured with the transvaginal probe at 16–18 gestational weeks with a length of cervix > 25 mm being the adequate cervical length group (CL > 25 mm, n = 238); a short cervix with a length of cervix ≤ 25 mm, being the short-cervix group (≤ 25 mm, n = 93).

In the 3rd cohort, 93 women with a short cervix were grouped depending on the PTB prevention modes: progesterone-only group (POG, n = 70) and progesterone-pessary group (PPG, n = 23). Micronized progesterone vaginal capsules (200 mg/daily) were prescribed to all pregnant women with an approved diagnosis for a short cervix (≤ 25 mm) by USCL assessment, and the treatment continued up to the 34th week of gestation following the current recommendations of the Healthcare Ministry of the Russian Federation (“Preterm birth” No. 15–4/10/2–9480, 17.12. 2013).

Application of a pessary was the patient’s choice. In most cases, the pessaries were placed between 20 and 28 weeks of gestation and were inserted after USCL assessment. In three patients pessaries started to be used at 19, 29, and 30 weeks of gestation. Pessaries remained in place up to the beginning of the spontaneous PTB or 37 weeks of gestation. 20 women were given Arabin pessaries (Dr. Arabin GmbH & Co. KG**,** Witten, Germany) and 3 patients—Yunona (Simurg, Vitebsk, Belarus) pessaries. There were no other additional treatments or other activities concerning PTB prevention.

### Demographic and obstetric data

Main demographic parameters including age, nationality, residency, education status (higher—university graduates; professional—education in specialized schools and colleges; secondary—general secondary education), smoking, prior reproductive (parity) history and the interval between cervical conization and conception were documented. Newborns in two women were fatal outcomes. Data concerning the use of antibiotics to prevent intrauterine infection and other therapeutic agents to improve neonatal outcomes (antenatal corticosteroids and magnesium sulfate) were not included because of their rarity. Results concerning newborns were not included in this study either.

#### Cervical condition assessment methods

Data concerning gynecological checkups of the condition of the cervix, including colposcopy and other laboratory methods, were not the scope of this study*.* Only the results of the cervical conization size and transvaginal ultrasound cervical length measurements were analyzed.

### Electrosurgical excision of the cervical lesions and cervical conization size assessment

Electrosurgical excision of the cervical lesions was performed with the Electrosurgical unit ESHF-80-03-(FOTEK, Ekaterinburg, Russia) using cone-shaped and loop probes. All surgical procedures were defined as cervical conization. The cervical conization size (CCS) or volume of the excised cervix (tissue specimen) was measured according to our previously described technique^67^. The measurement system included a syringe containing water 10 ml (see Fig. 2a–d). The tissue specimen was immediately inserted into the syringe, which sinks into the water while the level of the water is rising; so, the exact volume of the specimen defined as the cervical conization size CCS in cm^3^ was estimated.

### Transvaginal ultrasound cervical length measurement in non-pregnant and pregnant women

The USCL of all patients was evaluated before and one month after cervical conization. We used the Siemens Sonoline G 60 (Siemens Healthcare GmbH, Erlangen, Germany) intravaginal probe with 6.5 MHz in two-dimensional modes. All measurements were performed during the follicular phase of the women’s menstrual cycle, repeated three times and their average value recorded. USCL was performed in the 16–18 week period of the pregnancy. The procedure was done following the technique described by Iams et al.^6^, and according to recommendations^5,9^, the measurements being repeated three times. Subsequently, the average value was recorded. An USCL of > 25 mm was considered to be of adequate cervical length, whereas a cervical length of ≤ 25 mm was defined as a short cervix.

### Obstetrical factors

The parity status (nulliparous or parous), the childbirth mode (Cesarean section or vaginal delivery), the amniotic membrane rupture rate and the delivery time of the registered active labour period with regular contractions more frequent than every 5 min and a dilated cervix of 3 cm or more^69^, were analyzed as the obstetrical factors.

### The final pregnancy endpoints

The duration (length) of pregnancy in weeks with survival curve analysis and the rate of spontaneous preterm birth (< 37 weeks of gestation) were considered as the final endpoints of the study.

### Statistical analysis

All medical records were retrieved from the database of the outpatient unit, depersonalized, and collected in Excel tables for further statistical analysis which was performed using the Graph Pad Prism. 8.0.2 program. The distribution normality of variables was achieved using D'Agostino & Pearson test and the QQ plots. Appropriate statistical tests with alpha = 0.05 were chosen accordingly to variable distribution type. A one-way analysis of variance (ANOVA) with Tukey’s multiple comparisons test was used to compare two groups, including baseline with follow-up, with twice performed repeated measurements. A two-way ANOVA with Bonferroni’s multiple comparisons test was applied for repeated measurements performed > than twice. The Mann–Whitney test (two-tailed) was used when variables did not pass normality tests. Comparisons of the pregnancy prolongation curves with proportions of prolonged pregnancies between 26 and 41 weeks of gestation and the childbirth duration (1–17 h) with the proportions of delivered women were performed using survival trend analysis with the Logrank (Mantel Cox), and Gehan–Breslow–Wilcoxon tests. The relative risk (RR) and odds ratios (OR) were calculated using the two-sided Fisher's exact test with confidential intervals computed with the Koopman asymptotic score for RR and the Baptista-Pike method for OR, as well as sensitivity and specificity, analyzed with the Wilson/Brown method. Results were described and presented as the mean values and proportions (percentages, %) with standard deviation (SD) or 95% confidential intervals (CI) of the mean, and also as box and whiskers with min to max or 1–99 percentiles, where two-sided P-value < 0.05 was considered as significant.

### Consent for publication

Depersonalized medical records from the outpatient unit of the Obstetric & Gynecology department of the Faculty of Medicine at Moscow State University of Medicine and Dentistry (named after A.I.Evdokimov) were collected and retrospectively analyzed. Initially, individual personal data by GPR guidelines with signed informed consent were received from all patients, the moment they underwent diagnostic and therapeutic interventions. No direct medical interventions were performed during this retrospective study.

### Duplicate publication statement

In this study, the results of a 10-year (2006–2016) practical undertaking were retrospectively analyzed. Therefore, some preliminary results have already been published in Russian journals: the results for the period 2007–2010 with the number of patients (n = 150)^a^; (2007–2011, n = 316)^b^; (2006–2014, n = 314)^c^. In these publications, different aspects of this subject matter were analyzed aiming to establish the relative volume of the cervix after conization and its impact on women’s reproductive health, but not the new concept presented in this manuscript and nor were all materials collected in this study (2006–2016 period, n = 621) published in any previous publication. We have cited the previously published correspondence with the description of the CCS assessment and we did not use any original figures from previous publications. The principles of the technique were presented in Fig. 2, by new drawings, acknowledging the painter. Prior publications: (a) Firichenko, S.V. et al. Electrosurgical cervical excision, and preterm delivery risk. Russian J. of Hum. Reprod. (4), 95–99, (2012), [Rus.]; (b) Firichenko, S.V. et al. Electrosurgical excision of the uterine cervix and the reproductive function. Prob. of Gyn. Obstet. & Perinat. **11** (6), 19–25, (2012), [Rus.]; (c) Firichenko, S.V. The reproductive function maintaining of women with cervical premalignant conditions. D.Sc. thesis, Moscow State University of Medicine and Dentistry named after A.I.Evdokimov, Moscow, 294 p. (2017), [Rus.].

## Supplementary Information

Supplementary Information.

## Data Availability

All relevant data are in the paper, and the authors can make materials available on request.
