# Supplementary material for: The impact of cervical conization size with subsequent cervical length changes on preterm birth rates in asymptomatic singleton pregnancies
Source: Sci Rep. 2021 Oct 5;11:19703. doi: 10.1038/s41598-021-99185-0 (PMC8492699; doi:10.1038/s41598-021-99185-0)
Supplement: Supplementary file 1 — Supplementary Information. [file 41598_2021_99185_MOESM1_ESM.pdf]

Supplementary Table 1. Demographic, obstetric, and pregnancy outcome parameters with values of mean and standard deviation (SD), 95% lower/upper confidential intervals (CI) of the mean in the 2nd cohort: Adequate Cervix Length (CL>25mm) vs. Short Cervix (CL≤25mm), groups. P-values were achieved using the Mann-Whitney two-tailed t-test with Alpha = 0.05.

Parameters	Groups				P values
	CL>25mm, n=238		CL≤25 mm, n=93		
	Mean, SD	95% CI	Mean, SD	95% CI	
Age, years	28.28±5.74	27.55/29.02	27.40±5.72	26.22/28.58	NS
Moscow residency, %	0.92±0.26	0.89/0.96	0.92±0.26	0.87/0.98	NS
Slavic ethnicity, %	0.87±0.34	0.83/0.91	0.86±0.35	0.79/0.93	NS
Higher education, %	0.32±0.49	0.26/0.38	0.31±0.47	0.22/0.41	NS
Professional education, %	0.38±0.49	0.32/0.44	0.38±0.49	0.28/0.48	NS
Secondary education, %	0.30±0.46	0.27/0.29	0.31±±0.46	0.26/0.29	NS
Smoking population, %	0.09±0.29	0.05/0.13	0.09±0.28	0.03/0.14	NS
Conization-pregnancy interval, months	12.28±4.61	11.69/12.87	14.30±6.61	12.94/15.66	0.0179
Cesarean section, %	0.18±0.39	0.13/0.23	0.23±0.42	0.14/0.31	NS
Vaginal birth, %	0.82±0.39	0.77/0.87	0.77±0.04	0.69/0.86	NS
Spontaneous amniotic membrane ruptures, %	0.12±0.32	0.08/0.16	0.15±0.36	0.08/0.22	NS

Supplementary Table 2. Demographic, obstetric, and pregnancy outcome parameters with values of mean and standard deviation (SD), 95% lower/upper confidential intervals (CI) of the mean in the 3rd cohort: Progesterone-only (POG) vs. Progesterone-Pessary (PPG), groups. P-values were achieved using the Mann-Whitney two-tailed t-test with Alpha = 0.05.

Parameters	Groups				P values
	POG, n=70		PPG, n=23		
	Mean, SD	95% CI	Mean, SD	95% CI	
Age, years	27.59±5.68	26.23/28.94	26.83±5.94	24.26/29.40	NS
Moscow residency, %	0.93±0.26	0.87/0.99	0.91±0.29	0.79/0.99	NS
Slavic ethnicity, %	0.86±0.35	0.77/0.94	0.87±0.34	0.72/0.99	NS
Higher education, %	0.30±0.4	0.19/0.41	0.35±0.49	0.14/0.41	NS
Professional education, %	0.37±0.49	0.25/0.49	0.39±0.50	0.17/0.61	NS
Secondary education, %	0.31±0.47	0.26/0.29	0.30±±0.47	0.24/0.29	NS
Smoking population, %	0.09±0.28	0.02/0.15	0.09±0.29	0.04/0.21	NS
Conization-pregnancy interval, months	14.09±6.9	12.44/15.73	14.96±5.71	12.49/17.43	NS
Cesarean section, %	0.24±0.43	0.14/0.35	0.17±0.39	0.01/0.34	NS
Vaginal birth, %	0.76±0.43	0.65/0.86	0.83±0.39	0.66/0.99	NS
Spontaneous amniotic membrane ruptures, %	0.14±0.35	0.06/0.23	0.17±0.39	0.01/0.34	NS
